# 5-(2-Phenyl­ethyn­yl)isobenzofuran-1,3-dione

**DOI:** 10.1107/S1600536808028407

**Published:** 2008-10-25

**Authors:** Mei-Jia Yang, Jian Men, Xiao-Yan Ma, Shi-Xu Yi, Guo-Wei Gao

**Affiliations:** a College of Chemistry, Sichuan University, Chengdu 610064, People’s Republic of China; b College of Materials and Chemical Engineering, Chengdu University of Technology, Chengdu 610059, People’s Republic of China

## Abstract

The title compound, C_16_H_8_O_3_, was synthesized by the Pd-coupling reaction of phenyl­acetyl­ene with 4-bromo­phthalic anhydride. The phenyl and isobenzofurane rings are nearly coplanar, forming a dihedral angle of 6.70 (10)°. In the crystal structure, centrosymmetrically related mol­ecules are linked into dimers by C—H⋯O hydrogen bonds.

## Related literature

For a general background to the synthesis and applications of the title compound, see: Hergenrother & Smith (1994 ▶, 1996 ▶); Takekoshi & Terry (1994 ▶); Urazoe *et al.* (2005 ▶); Urazoe & Mori (2006 ▶). For the properties of polyimides, see: Feger *et al.* (1989 ▶); Ghosh & Mittal (1996 ▶). For the crystal structure of related compounds, see: Wright & Schorzman (2000 ▶).
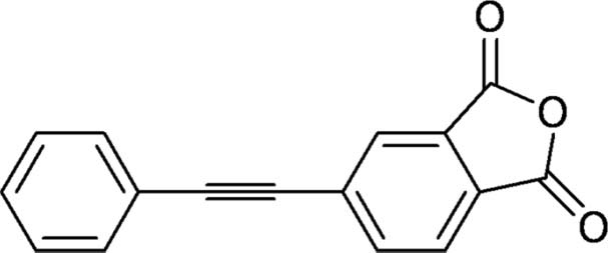


## Experimental

### 

#### Crystal data

C_16_H_8_O_3_
*M* *_r_* = 248.22
Triclinic, 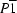

*a* = 6.998 (3) Å
*b* = 7.518 (3) Å
*c* = 11.683 (4) Å
α = 89.06 (2)°
β = 79.31 (3)°
γ = 81.04 (2)°
*V* = 596.6 (4) Å^3^
*Z* = 2
Mo *K*α radiation
μ = 0.10 mm^−1^
*T* = 294 (2) K
0.50 × 0.40 × 0.22 mm

#### Data collection

Enraf–Nonius CAD4 diffractometer
Absorption correction: none
2227 measured reflections
2198 independent reflections
1105 reflections with *I* > 2σ(*I*)
*R*_int_ = 0.007
3 standard reflections every 100 reflections intensity decay: 1.8%

#### Refinement

*R*[*F* ^2^ > 2σ(*F* ^2^)] = 0.064
*wR*(*F* ^2^) = 0.158
*S* = 1.09
2198 reflections
173 parameters
H-atom parameters constrained
Δρ_max_ = 0.21 e Å^−3^
Δρ_min_ = −0.21 e Å^−3^

### 

Data collection: *DIFRAC* (Gabe & White, 1993 ▶); cell refinement: *DIFRAC*; data reduction: *NRCVAX* (Gabe *et al.*, 1989 ▶); program(s) used to solve structure: *SHELXS97* (Sheldrick, 2008 ▶); program(s) used to refine structure: *SHELXL97* (Sheldrick, 2008 ▶); molecular graphics: *ORTEP-3 for Windows* (Farrugia, 1997 ▶); software used to prepare material for publication: *SHELXL97*.

## Supplementary Material

Crystal structure: contains datablocks global, I. DOI: 10.1107/S1600536808028407/rz2241sup1.cif

Structure factors: contains datablocks I. DOI: 10.1107/S1600536808028407/rz2241Isup2.hkl

Additional supplementary materials:  crystallographic information; 3D view; checkCIF report

## Figures and Tables

**Table 1:** Hydrogen-bond geometry (Å, °)

*D*—H⋯*A*	*D*—H	H⋯*A*	*D*⋯*A*	*D*—H⋯*A*
C16—H16⋯O2^i^	0.93	2.56	3.436 (5)	158

Symmetry code: (i) 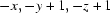.

**Fig. 1.**
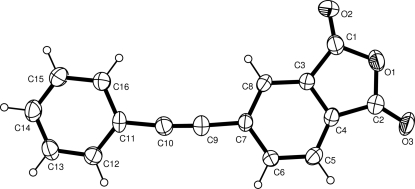
The molecular structure of the title compound, with displacement ellipsoids drawn at the 30% probability level.

## supplementary crystallographic information

### Comment

Polyimides are well known for possessing excellent thermal and oxidative stability, as well as excellent mechanical properties (Ghosh & Mittal, 1996; Feger *et al.*,1989). The title compound is used as terminal endcapping agent which imparts thermal curability, thermal resistance and solvent resistance to polyimide (Hergenrother & Smith, 1994, 1996; Takekoshi & Terry, 1994). Further, various types of method for producing 4-phenylethynylphthalic anhydride have been described (Urazoe *et al.*, 2005; Urazoe & Mori, 2006). We report here the crystal sturcture of the title compound.

In the molecule of the title compound (Fig. 1) the phenyl and isobenzofurane rings are nearly coplanar, making a dihedral angle of 6.70 (10)°. Bond distances of the ethyne chain show similar values to those in C_22_H_13_O_2_N (Wright & Schorzman, 2000), with the C7—C9, C9—C10 and C10—C11 distances of 1.418 (4), 1.203 (4) and 1.429 (4) Å, respectively. The bond angles within the ethyne chain are slightly bent, with the C7—C9—C10 and C9—C10—C11 angles of 176.3 (4) and 173.6 (4)°, respectively. The isobenzofurane ring is flat, atoms C2 deviating only by 0.027 (5) Å from the mean plane. In the crystal structure, centrosymmetrically related molecules are linked into dimers by intermolecular C—H···O hydrogen interactions (Table 1).

### Experimental

4-Bromophthalic anhydride (5.00 g, 22.0 mmol), phenylacetylene (2.69 g, 26.4 mmol), PdCl_2_(PPh_3_)_2_ (0.11 g, 0.157 mmol), and PPh_3_ (0.22 g, 0.840 mmol) were dissolved in 40 ml of dry NEt_3_ under argon, and the mixture was heated to 333 K. Then CuI (0.10 g, 0.524 mmol) was added and the solution was stirred at 353 K for 12 h. The precipitated triethylammonium bromide was separated after cooling and the solvent was evaporated. The residue was recrystallized from toluene/n-hexane (1:1 v/v) twice to give 4-phenylethynylphthalic anhydride as pale yellow crystals (yield 83.6%; m.p. 424–425 K). Colourless crystals suitable for X-ray analysis were obtained by slow evaporation of an acetic anhydride solution at room temperature.

### Refinement

H atoms were positioned geometrically (C—H = 0.93 Å) and refined using a riding model, with *U*_iso_(H) = 1.2*U*_eq_ (C).

### Crystal data

**Table:**

C_16_H_8_O_3_	*Z* = 2
*M**_r_* = 248.22	*F*(000) = 256
Triclinic, *P*1	*D*_x_ = 1.382 Mg m^−^^3^
Hall symbol: -P 1	Mo *K*α radiation, λ = 0.71073 Å
*a* = 6.998 (3) Å	Cell parameters from 25 reflections
*b* = 7.518 (3) Å	θ = 4.5–11.8°
*c* = 11.683 (4) Å	µ = 0.10 mm^−^^1^
α = 89.06 (2)°	*T* = 294 K
β = 79.31 (3)°	Block, colourless
γ = 81.04 (2)°	0.50 × 0.40 × 0.22 mm
*V* = 596.6 (4) Å^3^	

### Data collection

**Table:**

Enraf–Nonius CAD4 diffractometer	*R*_int_ = 0.007
Radiation source: fine-focus sealed tube	θ_max_ = 25.5°, θ_min_ = 2.7°
graphite	*h* = −8→8
ω/2θ scans	*k* = −3→9
2227 measured reflections	*l* = −14→14
2198 independent reflections	3 standard reflections every 100 reflections
1105 reflections with *I* > 2σ(*I*)	intensity decay: 1.8%

### Refinement

**Table:**

Refinement on *F*^2^	Secondary atom site location: difference Fourier map
Least-squares matrix: full	Hydrogen site location: inferred from neighbouring sites
*R*[*F*^2^ > 2σ(*F*^2^)] = 0.064	H-atom parameters constrained
*wR*(*F*^2^) = 0.158	*w* = 1/[σ^2^(*F*_o_^2^) + (0.0535*P*)^2^] where *P* = (*F*_o_^2^ + 2*F*_c_^2^)/3
*S* = 1.09	(Δ/σ)_max_ < 0.001
2198 reflections	Δρ_max_ = 0.22 e Å^−^^3^
173 parameters	Δρ_min_ = −0.21 e Å^−^^3^
0 restraints	Extinction correction: SHELXL97 (Sheldrick, 1997), Fc^*^=kFc[1+0.001xFc^2^λ^3^/sin(2θ)]^-1/4^'
Primary atom site location: structure-invariant direct methods	Extinction coefficient: 0.014 (4)

### Special details

**Table:**

Geometry. All e.s.d.'s (except the e.s.d. in the dihedral angle between two l.s. planes) are estimated using the full covariance matrix. The cell e.s.d.'s are taken into account individually in the estimation of e.s.d.'s in distances, angles and torsion angles; correlations between e.s.d.'s in cell parameters are only used when they are defined by crystal symmetry. An approximate (isotropic) treatment of cell e.s.d.'s is used for estimating e.s.d.'s involving l.s. planes.
Refinement. Refinement of *F*^2^ against ALL reflections. The weighted *R*-factor *wR* and goodness of fit *S* are based on *F*^2^, conventional *R*-factors *R* are based on *F*, with *F* set to zero for negative *F*^2^. The threshold expression of *F*^2^ > 2σ(*F*^2^) is used only for calculating *R*-factors(gt) *etc*. and is not relevant to the choice of reflections for refinement. *R*-factors based on *F*^2^ are statistically about twice as large as those based on *F*, and *R*- factors based on ALL data will be even larger.

### Fractional atomic coordinates and isotropic or equivalent isotropic displacement parameters (Å^2^)

**Table:**

	*x*	*y*	*z*	*U*_iso_*/*U*_eq_	
O1	0.5201 (3)	0.1623 (3)	0.3262 (2)	0.0689 (8)	
O2	0.3130 (4)	0.3047 (4)	0.4814 (2)	0.0912 (10)	
O3	0.6535 (3)	0.0590 (3)	0.1454 (2)	0.0814 (9)	
C1	0.3406 (5)	0.2634 (5)	0.3814 (4)	0.0655 (11)	
C2	0.5145 (5)	0.1372 (5)	0.2089 (4)	0.0621 (10)	
C3	0.2138 (5)	0.2980 (4)	0.2931 (3)	0.0484 (9)	
C4	0.3202 (4)	0.2205 (4)	0.1897 (3)	0.0489 (9)	
C5	0.2391 (5)	0.2304 (4)	0.0913 (3)	0.0604 (10)	
H5	0.3096	0.1777	0.0216	0.072*	
C6	0.0505 (5)	0.3205 (4)	0.0986 (3)	0.0570 (10)	
H6	−0.0073	0.3273	0.0329	0.068*	
C7	−0.0562 (4)	0.4021 (4)	0.2025 (3)	0.0505 (9)	
C8	0.0267 (5)	0.3885 (4)	0.3023 (3)	0.0521 (9)	
H8	−0.0430	0.4392	0.3728	0.062*	
C9	−0.2470 (5)	0.5020 (4)	0.2070 (3)	0.0607 (10)	
C10	−0.4084 (5)	0.5899 (4)	0.2170 (3)	0.0603 (10)	
C11	−0.6025 (4)	0.6902 (4)	0.2426 (3)	0.0534 (9)	
C12	−0.7133 (5)	0.7369 (4)	0.1575 (3)	0.0594 (10)	
H12	−0.6615	0.7056	0.0801	0.071*	
C13	−0.9015 (5)	0.8304 (5)	0.1881 (4)	0.0667 (11)	
H13	−0.9752	0.8638	0.1305	0.080*	
C14	−0.9807 (5)	0.8744 (5)	0.3007 (4)	0.0697 (11)	
H14	−1.1088	0.9354	0.3204	0.084*	
C15	−0.8719 (5)	0.8288 (5)	0.3852 (3)	0.0726 (11)	
H15	−0.9272	0.8589	0.4624	0.087*	
C16	−0.6828 (5)	0.7396 (4)	0.3586 (3)	0.0647 (11)	
H16	−0.6088	0.7123	0.4167	0.078*	

### Atomic displacement parameters (Å^2^)

**Table:**

	*U*^11^	*U*^22^	*U*^33^	*U*^12^	*U*^13^	*U*^23^
O1	0.0505 (16)	0.0776 (17)	0.076 (2)	0.0049 (13)	−0.0171 (14)	0.0015 (15)
O2	0.081 (2)	0.137 (3)	0.0513 (18)	0.0076 (17)	−0.0214 (16)	−0.0083 (17)
O3	0.0510 (16)	0.0859 (19)	0.095 (2)	0.0177 (14)	−0.0024 (15)	−0.0271 (16)
C1	0.054 (2)	0.075 (3)	0.065 (3)	0.000 (2)	−0.011 (2)	0.001 (2)
C2	0.056 (3)	0.058 (2)	0.070 (3)	−0.0026 (19)	−0.010 (2)	−0.004 (2)
C3	0.046 (2)	0.049 (2)	0.049 (2)	−0.0002 (16)	−0.0090 (18)	−0.0020 (17)
C4	0.0405 (19)	0.047 (2)	0.056 (2)	0.0001 (15)	−0.0068 (18)	−0.0085 (17)
C5	0.057 (2)	0.061 (2)	0.058 (2)	0.0036 (18)	−0.007 (2)	−0.0159 (19)
C6	0.053 (2)	0.064 (2)	0.051 (2)	0.0041 (18)	−0.0135 (18)	−0.0154 (18)
C7	0.041 (2)	0.050 (2)	0.057 (2)	0.0056 (16)	−0.0105 (18)	−0.0051 (17)
C8	0.044 (2)	0.058 (2)	0.048 (2)	0.0031 (17)	−0.0006 (18)	−0.0095 (17)
C9	0.054 (2)	0.059 (2)	0.068 (3)	−0.004 (2)	−0.013 (2)	−0.0032 (19)
C10	0.061 (2)	0.058 (2)	0.064 (3)	−0.006 (2)	−0.019 (2)	−0.0029 (19)
C11	0.036 (2)	0.0445 (19)	0.076 (3)	0.0035 (16)	−0.009 (2)	−0.0023 (18)
C12	0.058 (2)	0.058 (2)	0.061 (2)	−0.0038 (18)	−0.012 (2)	−0.0039 (18)
C13	0.057 (2)	0.063 (2)	0.085 (3)	−0.005 (2)	−0.028 (2)	−0.003 (2)
C14	0.055 (2)	0.063 (2)	0.083 (3)	0.0072 (19)	−0.006 (2)	−0.014 (2)
C15	0.074 (3)	0.070 (3)	0.066 (3)	0.011 (2)	−0.009 (2)	−0.016 (2)
C16	0.061 (2)	0.067 (3)	0.065 (3)	0.004 (2)	−0.020 (2)	−0.008 (2)

### Geometric parameters (Å, °)

**Table:**

O1—C2	1.395 (4)	C8—H8	0.9300
O1—C1	1.411 (4)	C9—C10	1.203 (4)
O2—C1	1.186 (4)	C10—C11	1.429 (4)
O3—C2	1.187 (4)	C11—C12	1.379 (5)
C1—C3	1.476 (5)	C11—C16	1.399 (4)
C2—C4	1.461 (4)	C12—C13	1.379 (4)
C3—C8	1.365 (4)	C12—H12	0.9300
C3—C4	1.381 (4)	C13—C14	1.354 (4)
C4—C5	1.369 (4)	C13—H13	0.9300
C5—C6	1.375 (4)	C14—C15	1.363 (5)
C5—H5	0.9300	C14—H14	0.9300
C6—C7	1.397 (4)	C15—C16	1.370 (4)
C6—H6	0.9300	C15—H15	0.9300
C7—C8	1.391 (4)	C16—H16	0.9300
C7—C9	1.418 (4)		
			
C2—O1—C1	109.3 (3)	C3—C8—H8	121.2
O2—C1—O1	121.4 (4)	C7—C8—H8	121.2
O2—C1—C3	131.4 (4)	C10—C9—C7	176.3 (4)
O1—C1—C3	107.2 (3)	C9—C10—C11	173.6 (4)
O3—C2—O1	120.3 (3)	C12—C11—C16	119.3 (3)
O3—C2—C4	132.2 (4)	C12—C11—C10	122.3 (3)
O1—C2—C4	107.5 (3)	C16—C11—C10	118.3 (3)
C8—C3—C4	122.4 (3)	C11—C12—C13	119.6 (3)
C8—C3—C1	130.3 (3)	C11—C12—H12	120.2
C4—C3—C1	107.3 (3)	C13—C12—H12	120.2
C5—C4—C3	120.6 (3)	C14—C13—C12	121.0 (4)
C5—C4—C2	130.8 (3)	C14—C13—H13	119.5
C3—C4—C2	108.7 (3)	C12—C13—H13	119.5
C4—C5—C6	118.1 (3)	C13—C14—C15	119.7 (4)
C4—C5—H5	121.0	C13—C14—H14	120.2
C6—C5—H5	121.0	C15—C14—H14	120.2
C5—C6—C7	121.6 (3)	C14—C15—C16	121.3 (4)
C5—C6—H6	119.2	C14—C15—H15	119.3
C7—C6—H6	119.2	C16—C15—H15	119.3
C8—C7—C6	119.8 (3)	C15—C16—C11	119.0 (3)
C8—C7—C9	119.4 (3)	C15—C16—H16	120.5
C6—C7—C9	120.8 (3)	C11—C16—H16	120.5
C3—C8—C7	117.6 (3)		
			
C2—O1—C1—O2	−178.4 (4)	C2—C4—C5—C6	−179.3 (3)
C2—O1—C1—C3	1.8 (4)	C4—C5—C6—C7	0.7 (5)
C1—O1—C2—O3	178.1 (3)	C5—C6—C7—C8	−1.8 (5)
C1—O1—C2—C4	−2.1 (4)	C5—C6—C7—C9	176.9 (3)
O2—C1—C3—C8	0.0 (7)	C4—C3—C8—C7	−0.4 (5)
O1—C1—C3—C8	179.7 (3)	C1—C3—C8—C7	179.1 (3)
O2—C1—C3—C4	179.5 (4)	C6—C7—C8—C3	1.6 (5)
O1—C1—C3—C4	−0.8 (4)	C9—C7—C8—C3	−177.2 (3)
C8—C3—C4—C5	−0.7 (5)	C16—C11—C12—C13	−0.4 (5)
C1—C3—C4—C5	179.7 (3)	C10—C11—C12—C13	178.6 (3)
C8—C3—C4—C2	179.1 (3)	C11—C12—C13—C14	−1.2 (5)
C1—C3—C4—C2	−0.4 (4)	C12—C13—C14—C15	1.3 (6)
O3—C2—C4—C5	1.1 (7)	C13—C14—C15—C16	0.3 (6)
O1—C2—C4—C5	−178.6 (3)	C14—C15—C16—C11	−1.8 (6)
O3—C2—C4—C3	−178.7 (4)	C12—C11—C16—C15	1.9 (5)
O1—C2—C4—C3	1.5 (4)	C10—C11—C16—C15	−177.2 (3)
C3—C4—C5—C6	0.5 (5)		

### Hydrogen-bond geometry (Å, °)

**Table:**

*D*—H···*A*	*D*—H	H···*A*	*D*···*A*	*D*—H···*A*
C16—H16···O2^i^	0.93	2.56	3.436 (5)	158

Symmetry codes: (i) −*x*, −*y*+1, −*z*+1.
